# Bonded CFRP/Steel Systems, Remedies of Bond Degradation and Behaviour of CFRP Repaired Steel: An Overview

**DOI:** 10.3390/polym13091533

**Published:** 2021-05-10

**Authors:** Daniel Borrie, Saad Al-Saadi, Xiao-Ling Zhao, R. K. Singh Raman, Yu Bai

**Affiliations:** 1 Department of Civil Engineering, Monash University, Clayton, VIC 3800, Australia; xiaolin.zhao@unsw.edu.au (X.-L.Z.); yu.bai@monash.edu (Y.B.); 2 Department of Mechanical and Aerospace Engineering, Monash University, Clayton, VIC 3800, Australia; raman.singh@monash.edu; 3 School of Civil and Environmental Engineering, University of New South Wales, Sydney, NSW 2052, Australia; 4 Department of Chemical Engineering, Monash University, Clayton, VIC 3800, Australia

**Keywords:** CFRP, steel, bond behaviour, failure modes

## Abstract

This literature review has examined the use of FRP composite materials as a potential retrofitting technique for civil structures. Importantly, the various material properties, bond mechanisms, durability issues and fatigue resistance have been discussed. Studies exploring the performance of CFRP repaired steel have strongly indicated its potential as a rehabilitation material. These systems offer many improvements over the current bulky and less chemically resistant methods of bolting or welding steel plate patches. This review has established and highlighted the factors that affect CFRP/steel bond durability, namely surface preparation, curing, corrosion, fatigue loading, temperature and moisture ingress through studies that focus on their effect. These studies, however, often focus on a single influencing factor or design criteria. Only limited studies have investigated multiple parameters applied simultaneously, even though they commonly occur together in industrial practice. This review aimed to summarise the numerous influencing parameters to give a clearer understanding of the relevance of CFRP repaired steel structures.

## 1. Introduction

Large numbers of civil infrastructure, such as marine structures, mining equipment and bridge components, are becoming severely degraded. Steel components that were installed are aging towards the extremes of their design life. These steel sections, which are often dynamically loaded, are also commonly exposed to extreme weather conditions. Whether it is extremes in temperature, humidity, salty waters, or UV radiation exposure, these environments create susceptibility to corrosion. Corrosion causes progressive weakening of structural elements that can result in crack initiation [1]. When fatigue loading and corrosion are combined, the loss of strength and stiffness dramatically increases [2], potentially causing failure.

Traditional methods for retrofitting these aging structures include cutting out and replacing plating [3] or bolting or welding of steel plates to the damaged site [4,5]. However, these techniques introduce several drawbacks to the system when installed such as: welding’s poor fatigue performance and residual stress [6], loss of cross section from bolt holes, no reduction in corrosion threat, increased self-weight and increases in time and cost due to the skilled labour and techniques required to move, position and install these bulky and heavy plates [7,8]. The drawbacks of traditional techniques have created a growing demand for fibre reinforced polymer (FRP) applications. In fact, advanced composites have been in use since World War II [9], with applications in aerospace and military industries. Their high strength-to-weight ratios and weather resistance has more recently created interest in these materials for bridge repair and rehabilitation [10] during the late 1970s.

Early civil based FRP applications focused on concrete structures as the versatility, durability, and price of concrete have made it the worlds most used construction material [11]. Despite these advantages, concrete’s popularity inevitably means large quantities of civil infrastructure are deteriorating and require rebuilding or repair. This has inspired many investigations into FRP implementation to strengthen concrete structures [12,13,14,15,16].

For civil applications to steel, carbon fibres are more commonly used because of their superior stiffness which is more desirable to prevent fatigue cracking and uncontrollable vibrations and deflections. Carbon reinforced polymers’ (CFRPs) high stiffness unfortunately comes with a high production and material cost [17] compared to glass. However, if its use leads to a reduction in maintenance and repair, it may become more economical over time [18]. In fact, despite their drawback in material cost, CFRP’s low weight and simple installation can provide overall cost savings of approximately 17.5% [7] over traditional steel repairs.

With the increased interest in CFRP’s application to civil infrastructure, the understanding of these systems is continually progressing. Thus, many studies have been carried out to explore the durability of adhered CFRP under extreme environmental exposure and fatigue loading. Research on this topic has become very detailed in the past two decades, with a number of experimental investigations, as well as several books [19,20,21] and detailed literary summaries published [3,22,23,24,25].

In this review, the bond behaviour between CFRP and steel, and factors that affect the performance of bonded systems, are carefully described. Emphasis is places on the aspects of durability including, temperature, humidity and seawater as well as the influence of fatigue loading on adhered CFRP/steel. The proposed remedies for bond degradation such as material insulation, surface priming and adhesive modification have been discussed. In addition, the gaps in the current understanding of the CFRP repaired steel and their durability to environmental exposure are discussed and highlighted.

## 2. Bond Behaviour between CFRP and Steel

After showing great potential in strengthening concrete structures, FRP materials were subsequently trailed as a potential material for retrofitting steel elements. Unfortunately, metallic structural rehabilitation is not as widespread as with concrete because it poses a different and more challenging set of issues [26]. In theory, the strengthening material should have a stiffness equal to or exceeding the base material [27]. This makes a variety of FRP materials suitable for strengthening concrete or soft metals; however, for steel, stiffer materials, such as CFRP materials, are best. The following sections will therefore focus on CFRP adhesion to steel structures.

### 2.1. Bond Test Fabrication and Method

Composite materials are comparatively weak in compression and, as such, are commonly applied as tension member reinforcement. Commonly, CFRP patched steel systems are tested via static testing methods. These give an easy repeatable methodology for experimental data to be analysed. Steel is often patched in critically stressed tension regions [28]. This can be done with bonded or un-bonded systems, with un-bonded joints using clamps and their friction to support the metallic substrate [29,30,31]. Bonded joints are often considered a more effective patching technique as they create lower stress concentrations at the joint [32] and are easier to install, requiring less operating space.

Surface preparation for these bonded regions is critical in ensuring the quality of the bond. Three surface parameters, surface energy, chemical composition and surface roughness, all effect the quality of adhesion [33]. Chemical bond formation requires a chemically active surface that is free from contaminants [32]. Exposing a fresh chemically active surface requires surface abrasion. This can be done by hand, using an abrasive pad, sand paper, or grinding; however, these methods tend to create folds in the surface, which can trap contaminants and moisture [7]. It is commonly accepted that the most effective way to achieve a high energy surface, uniform roughness, and a chemically active surface is using grit-blasting [7,33,34,35], finished by wiping the surface with a chemical solvent before adhesives or primers are applied. A low viscosity adhesive can then fill the rough crevasses and cracks to provide the mechanism to transfer the load to the adhered CFRP. Poorly prepared surfaces can expect to undergo premature debonding, resulting in significantly reduced strengths.

**Figure 1:**
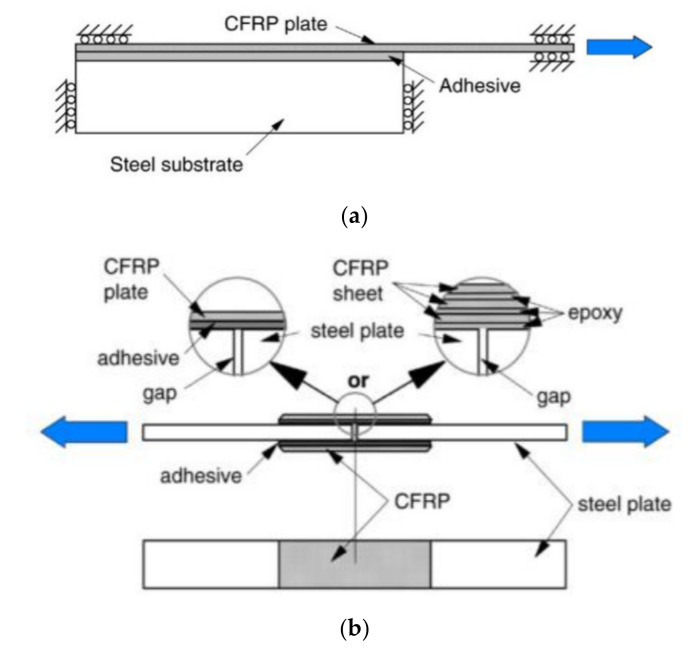
Test configurations of CFRP/steel joints (**a**) a single lap (single composite patch) shear joint. (**b**) a double lap shear joint [3]; used with permission from Engineering structures, Elsevier, 2021.

To examine the tensile bond strength of an adhered composite, several test configurations have been proposed [3]. However, in order to find the bond relationships under tension, two configurations are recommended and summarised in Figure 1. The configuration descriptions are as follows: 

Configuration (a): a single lap (single composite patch) shear joint where the loading is applied directly to the CFRP composite while the steel substrate remains fixed.

Configuration (b): a double lap shear joint where tensile load is applied (in opposite directions) to both ends of the steel substrate.

### 2.2. Failure Modes of CFRP/Steel Systems

The failure modes of tensile loaded, adhered CFRP to steel systems have been categorised by multiple authors into six failure modes [3,36]:

- (a) Steel and adhesive interface failure.
- (b) Cohesive failure (adhesive layer failure).
- (c) CFRP and adhesive interface failure.
- (d) CFRP delamination (separation of some carbon fibres from the resin matrix).
- (e) CFRP ruptures.
- (f) Steel yielding.

**Figure 2:**
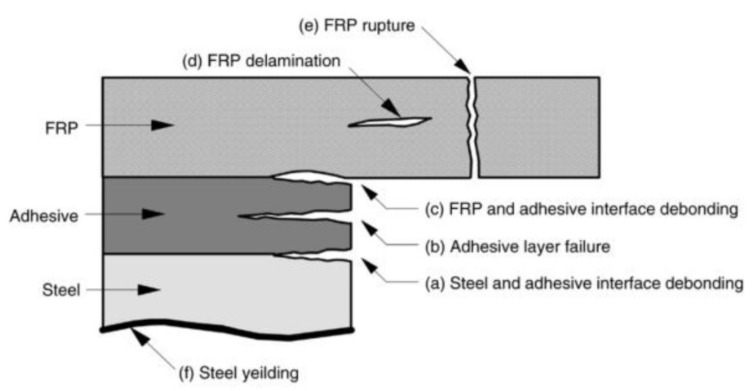
Schematic failure modes of adhered CFRP to steel joints [3,36], used with permission from Engineering structures, Elsevier, 2021.

Figure 2 shows a schematic representation of these failure modes within the bond joint. These failure modes rely heavily on several factors of the joint configuration, including the modulus, thickness, bond length and number of layers of CFRP, modulus, thickness and viscosity of the adhesive, as well as the thickness, yield strength and surface preparation of the metal substrate.

Commonly, the failure mode of normal modulus (NM) sheeting (240 GPa) is a mixed mode failure of (a) and (d) [37,38], whereas NM laminate (165 GPa) materials fail through cohesive failure (b) [39]. High modulus (HM) CFRP sheeting (640 GPa) fails via CFRP rupture (e) [38], with ultra-high modulus (UHM) laminates (460 GPa) tending to fail through CFRP rupture [40]. These highlight the different failure modes that occur as the modulus and type of CFRP change. Table 1 summarises the bond test methods and the highlights of previous studies.

### 2.3. Factors That Affect the Performance of Bonded CFRP/Steel Systems

Several important variables have been identified as common internal and external conditions that may influence bond durability of CFRP/steel joints. The details of a number of these and their subsequent investigations are outlined in the following sections.

#### 2.3.1. Adhesive Selection and Application

In addition to providing a medium for polymer bond, adhesives also provide protection to the fibres against environmental attack (insulation), as well as load-transfer between the composite and the substrate. Typical resins that are used in composite materials are epoxy, polyester, phenolic, or polyurethane resins [19], although epoxy adhesives are the most common when bonding fibre polymers to steel [7].

Adhesives require a curing or drying process in order to polymerise and harden. This curing process can be undertaken at ambient temperatures, which takes several days, or improved through elevated temperature curing. However, ambient temperature curing is best suited to large scale civil applications due to the simplicity, energy reduction and scale of the works. The alterations to this curing process have been heavily investigated already [41,42,43,44,45,46,47].

Adhesives have different chemical, physical and mechanical properties, but they should, when possible, remain compatible with the FRP’s resin [48]. Apart from the obvious cost considerations, several other adhesive features need to be considered before installation [20]:

- Tensile strength and modulus, to ensure the strength is sufficient and the stiffness is compatible with the FRP material.
- Shear strength and ductility, in order to provide the necessary load transfer capabilities, deformability, and toughness.
- Fatigue resistance, which is critical in tension members subjected to cyclic loads such as bridge trusses or transmission line poles in windy areas.
- Environmental durability, especially when repairing structures that operate in aggressive environments.
- Curing time and temperature, in order to attain the design strength rapidly and preferably without the need of artificial heating.
- Workability, as adhesives must be viscous enough to remain in place during bonding.
- Pot life, where higher values benefit constructability by facilitating installation over large areas.

Furthermore, the ability of adhesives to withstand extreme temperature exposures relies heavily on their physical property referred to as the glass transition temperature (Tg). When exposed to temperatures around or higher than the Tg, adhesives have a reduction in stiffness [49], resulting in lower bond strength, making them susceptible to high temperatures. There is no uniformly acceptable or appropriate adhesive for all applications for steel; hence, an appropriate evaluation needs to be made prior to implementation.

Ideally, adhesive layers should remain below 0.5 mm, with thin uniform layers being most desirable [50]. Thickness layers are commonly controlled by either weight compressing [47], rolling [51] or a customised device [52]. Adhesive thicknesses also play a key role in stress transfers and bond behaviours of FRP-patched steel [37,39,53]. Stress transfers between thicker adhesives can also decrease bond strengths and change failure modes from cohesion failure to delamination failure [39].

The failure mode and strength are also significantly affected by the adhesive ductility. Wu et al. [54] investigated UHM laminate bonded with multiple epoxy adhesives. It was found that as adhesive ductility increased failure modes shifted from cohesive failure to CFRP delamination.

As well as the ductility, the adhesive thickness plays a vital role in specimen failure mode. Xia and Teng [39] found that NM laminate bonds suffered cohesive failure for adhesive thicknesses of less than 2 mm and CFRP delamination for thicknesses greater than 2 mm. However, for similar CFRP laminates, Yu et al. [55] achieved constant cohesion failure for specimen with thicknesses of 1, 1.5, 2 and 3 mm.

#### 2.3.2. Galvanic Corrosion

Many steel structures requiring rehabilitation are located near sea waters, or in the case of bridges, have de-icing salts washed over them to prevent icy roads. Sea water and salt solutions are electrolytes that are highly corrosive for common engineering metals and alloys, such as steels. Glass fibres, being non-conductive, prevent galvanic corrosion in these electrolytes; CFRP however, is highly cathodic and possesses a considerable electrochemical difference with steel in these environments. Hence, it is highly likely that galvanic corrosion may occur when CFRP and steel are in contact with one another in the presence of a salt solution. This corrosion, if localised to pitting, can reduce the fatigue life of these structures inherently negating the advantages of the CFRP patch.

Torres-Acosta [56] produced a series of experiments studying the parameters that would promote galvanic corrosion of carbon fibre polymers and steel. The initial study used electrochemical cells of steel and pultruded CFRP rods in a variety of conditions. The steel was “as received” cleaned with acetone, with an epoxy capping. CFRP on the other hand, was either (1) “as received”, with an epoxy cap; (2) degraded by 2.5% of its surface area, with an epoxy cap; or (3) a free end rod with its end cut in direct contact with the mortar. It is understood that when epoxy adhesives are used, they reduce and often prevent corrosion, although 1% breaks in epoxy are not unreasonable [57], hence the use of the free ended rod. With the rods placed in the cell at 20–24 °C in wet/dry cycles for 460 days the highest galvanic corrosion density witnessed was 1.5 µA/cm^2^, equivalent to a steel loss of 0.018 mm/year. This rate is considered high and would reduce the service life of such members. Further findings included: mortars with no Cl^−^ had negligible corrosion rates, dry cycles had higher corrosion levels and also if a more active steel or an increase in the CFRP:steel (cathode:anode) ratio was used, corrosion rates would increase.

**Figure 3:**
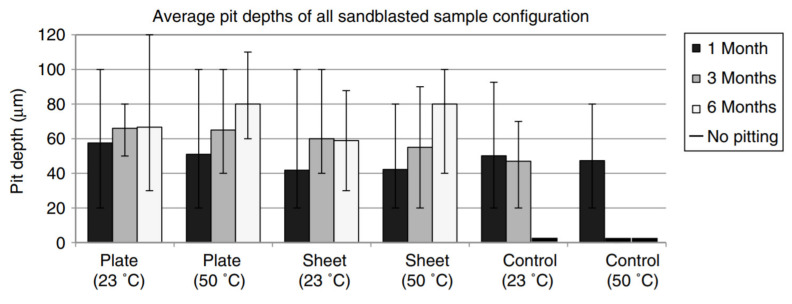
Pit depths of all sandblasted specimens for up to 6 months of submergence [58]; used with permission from Advances in Structural Engineering, SAGE, 2021.

Borrie et al. [58] studied the interactions of CFRP and steel in extreme weather conditions and the material characteristics in causing localised corrosion (e.g., pitting). Steel tiles placed in direct contact with different varieties of CFRP were exposed to 5% NaCl solutions at two temperatures for different durations. Two MBrace manufactured CFRP materials were used (i.e., high modulus CFRP sheeting reinforcement that was comprised of exposed, unidirectional fibres and a normal modulus laminate made from unidirectional carbon fibres embedded into a resin matrix). They found that most pits were within 20–50 µm; however, there were a few pits with depths in the range of 80–100 µm and occasionally, isolated pits were even deeper (160 µm). The average pit depths in Figure 3 show that the CFRP laminate patched specimens produced the largest pit depths (54.3 µm) among the patched samples. They reported that temperature had a limited effect on pit development during this short exposure period. An increase in temperature caused the plate and control specimens’ pit depths to decrease by 11.4% and 12.9%, respectively.

More directly, Tavakkolizadeh and Saadatmanesh [28] studied the potentiodynamic polarisation and galvanic corrosion of CFRP and steel, testing a total of 38 specimens with two environmental conditions and three thicknesses of epoxy coating the materials. Using de-icing salt solution or sea water, epoxy coatings varied from a thin coating to a saturated fibre coating and a large epoxy cover on the patched steel. Several important conclusions were made, including that, even with a thin layer of epoxy (0.1 mm), the galvanic coupling decreases four or five times more than that of samples with no epoxy. However, there is a twenty-one to twenty-three times decrease in corrosion rates as the epoxy thickens to 0.25 mm.

Due to the possibility of galvanic corrosion several chemical and physical protection barriers have been investigated to assess their ability to prevent corrosion fatigue. Chemical coatings, such as silane, are applied directly to steel surface as a primer before adhesive application. They are renowned for their corrosion protection, as well as their ability to form more primary bonds between adhesives and steel, in turn increasing bond strength. Sizemore et al. [50] found that a low percentage silane solution coating increased bond strength by 20%. With a stable life of 24 h and the ability to be sprayed or painted onto large surfaces, the solution can easily translate into field applications. In further studies under environmental exposure silane showed the ability to increase durability of CFRP to steel double lap joints [59]. Configurations involving silane primers had a maximum strength loss of 16% after 6 months exposure to saline solutions, whereas untreated specimens witnessed up to 60% loss of strength.

Epoxy resins and barrier protection materials are available, although if they are ignored, misapplied, or mechanically degraded it is understood from these studies that the CFRP, and steel, in contact and exposed to saline solutions (such as sea water) will create a favourable condition for galvanic corrosion. Corrosion rates have been chemically analysed however the localised galvanic corrosion levels, in terms of isolated pitting, are yet to be researched. This pitting may in fact cause regions of high stress concentration and may become the site of premature structural failure. This potentially accelerated failure may play a significant role in the life extension and durability of these systems, in turn becoming a significant factor in the use of CFRP repair methods.

#### 2.3.3. Sustained Loading

Structures constantly experience loading that contains the self-weight of their components. This service loading can make up a significant portion of the overall loading scheme. Sustained loading on CFRP/steel systems has also proven to alter the bond performance and strength depending on the severity of the load. The sustained loading is often described as a percentage of the ultimate load of the CFRP bond, allowing the severity of the load to be compared between configurations.

**Figure 4:**
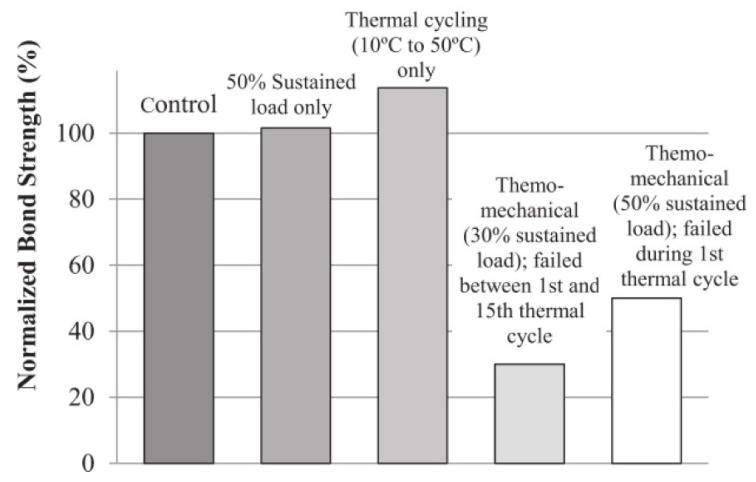
Bond strength behaviour of specimens under combined sustained load and thermal exposure [60]; used with permission from Composites Part B: Engineering, Elsevier, 2021.

Agarwal et al. [60] applied sustained loads of 30 and 50% of the ambient tested specimen strengths. It was found that specimens subjected to sustained loads for 21 days in isolation resulted in no significant reductions in static strength (Figure 4). However, when sustained load was combined with elevated temperatures from 10 to 50 °C, all specimens underwent failure before their allotted 108 thermo-cycles. It must be noted that the exposure temperature remained below the Tg of the adhesive, which is 62 °C.

Nguyen et al. [61,62,63] also investigated the effects of altering the service load on specimens while simultaneously exposing samples to extreme temperatures. The double lap joints prepared with normal modulus CFRP sheeting were subjected to increasing temperatures, from room temperature, while being subjected to sustained loads. Specimens were subjected to levels of sustained load and temperatures for 150 min before being tested under static tension. Specimens subjected to 80% of their ultimate strength and held at temperatures below the Tg of the epoxy managed to retain 100% of their static tensile performance. Those under small (20%) service loading at temperature above the Tg failed to survive their allotted service time. Thus, despite significantly high sustained loads, the bond performance was not compromised if temperatures remained below the Tg. In [47], the authors extended this investigation to include the influence of humidity with temperature cycling. Double lap joints were prepared with three layers of normal modulus CFRP sheeting. After curing, specimens were subjected to sustained loads while simultaneously being exposed to cyclical temperature changes between 20 and 50 °C at 90% humidity. All specimens cured at room temperature failed catastrophically after only 2 h of exposure when as little as 15% of the ultimate strength was applied as a sustained load.

The findings of these studies suggest that there is a certain sustained and stress level below which no damage is observed in the bond strength [64,65]. Hence the effect of sustained load on CFRP/steel systems is entirely dependent on the components of its configuration, the surrounding environment, and their interaction. If separately applied, sustained loading does not appear to affect the bond strength of CFRP/steel systems but can result in considerable reductions if applied in conjunction with other environmental or loading scenarios.

#### 2.3.4. Fatigue Loading

The majority of steel infrastructure requiring retrofitting undergo fatigue loading to some extent or another. Bridges for instance, with their long spans are prone to deflection and vibrations. Fatigue loading is often considered an important variable in affecting bond strength between FRP’s and steel.

Fatigue studies conducted on CFRP patched steel were designed to replicate the reinforcement of steel beams tension flanges [66]. One continuous length of steel was patched with CFRP laminates before being subjected to various fatigue load ranges. Initial debonding was witnessed at the CFRP ends before translating through the interface until mid-span. This progressive debonding caused a dramatic reduction in the stiffness of the specimens which was highlighted as a problem for progressive global failure. However, it is stated that to improve fatigue performance, joint design should be better optimised. In this study, several parameters were not ideal for greatest bond performance, such as grinding of steel surfaces and no control on the adhesive thickness which may have contributed to this debonding and subsequent lack of improvement.

One study focused on the impacts of tensile fatigue loading on normal modulus CFRP plate bonds with steel [67]. Double sided patched joints were loaded with one million fatigue cycles before being static tensile tested to failure. The fatigue load range was based on the tensile elastic limit of the samples. Double lap joint specimens that experienced fatigue loading above the lower sensitivity limit experienced some cracking from the central join. Overall, these specimens witnessed strength losses of 12–17% compared to those without fatigue.

**Figure 5:**
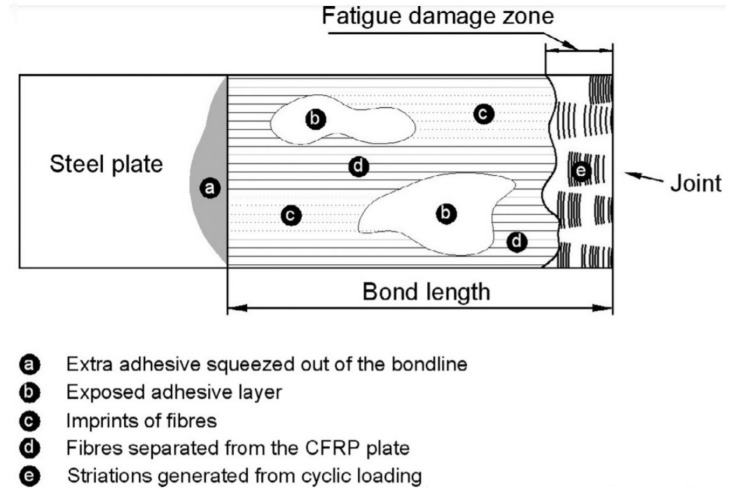
Microscopic scan locations on the fracture surface of the failed specimens and illustration of “fatigue damage zone” [68]; used with permission from Composites Part B: Engineering, Elsevier, 2021.

Furthermore, two studies conducted at Monash University explored fatigue effects on CFRP sheeting and laminates. Liu et al. [38] ran a series of fatigue-static tests on patched steel double lap-joints. For joints with stiffer high modulus sheeting, no fatigue damaged was witnessed even after 10 million loading cycles with stress ratios as high as 0.55. However, for specimens with normal modulus CFRP fatigue failure occurred when stress ratios exceeded 0.3, concluding that stiffer high modulus FRPs are better suited to cyclic loading scenarios. Wu et al. [68] found that fatigue loading had little effect on the specimen’s stiffness or strength when using ultra-high modulus (UHM) CFRP laminates. A maximum bond strength reduction of 4.7% was recorded after fatigue loading was applied. A “fatigue damage zone” was highlighted as the area closest to the joint that was affected by fatigue loading (Figure 5). In this case it was only approximately 1% of the bond length, confirming that small effect fatigue loading has on the bond strength and stiffness.

A number of these studies were concisely summarised by Zhao et al. [25]. In general, the better performance of high modulus materials over their normal modulus counterparts. However, none of the studies summarised or to that are known to the author have investigated the influence of fatigue in conjunction with environmental exposure and moisture. The damages seen through fatigue are likely to escalate in the presence of elevated temperatures or water saturation.

#### 2.3.5. Elevated Temperature

Elevated temperatures have proved to reduce curing times whilst increasing mechanical and physical properties of adhesives. However, high-temperature exposure can significantly alter bond performance if they are applied at the time of loading. Commonly, this temperature fluctuation is considered damaging due to the differing thermal coefficients of CFRP, adhesives and steel. Thermal coefficients of steel are approximately 10.8 × 106 /°C, whereas fibres are often considered zero, or potentially negative. This differential creates thermal stresses at the bonded surface, potentially causing permanent damage to the interface or the comparatively brittle adhesive.

Civil infrastructure exposed to summer environments and direct sunlight can reach service temperatures of up to, or beyond, 50 °C [47]. When adhesives, or resins of FRP composites, reach close to and above their Tg it leads to a reduction of mechanical properties and premature failure in bonded systems [69]. The Tg of many commonly used epoxies for FRP adhesion sit around 50 °C creating a severe risk of premature debonding and failure.

**Figure 6:**
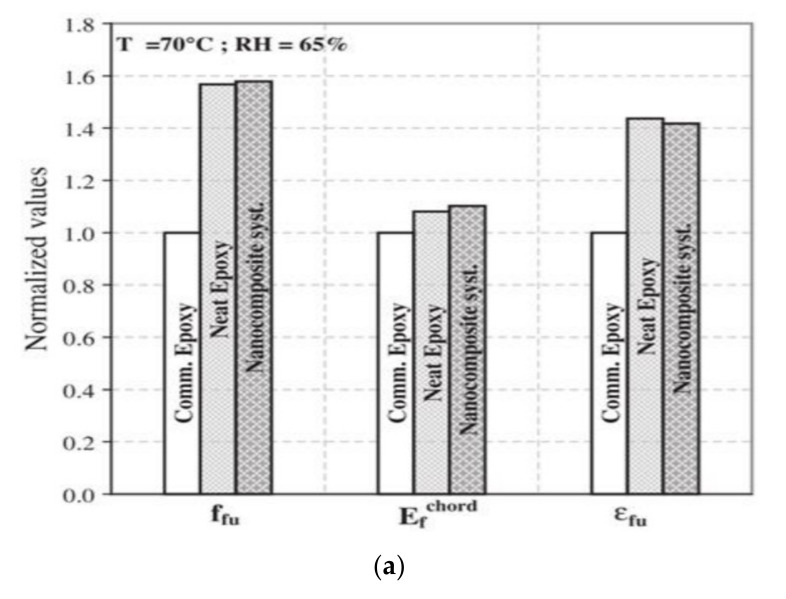

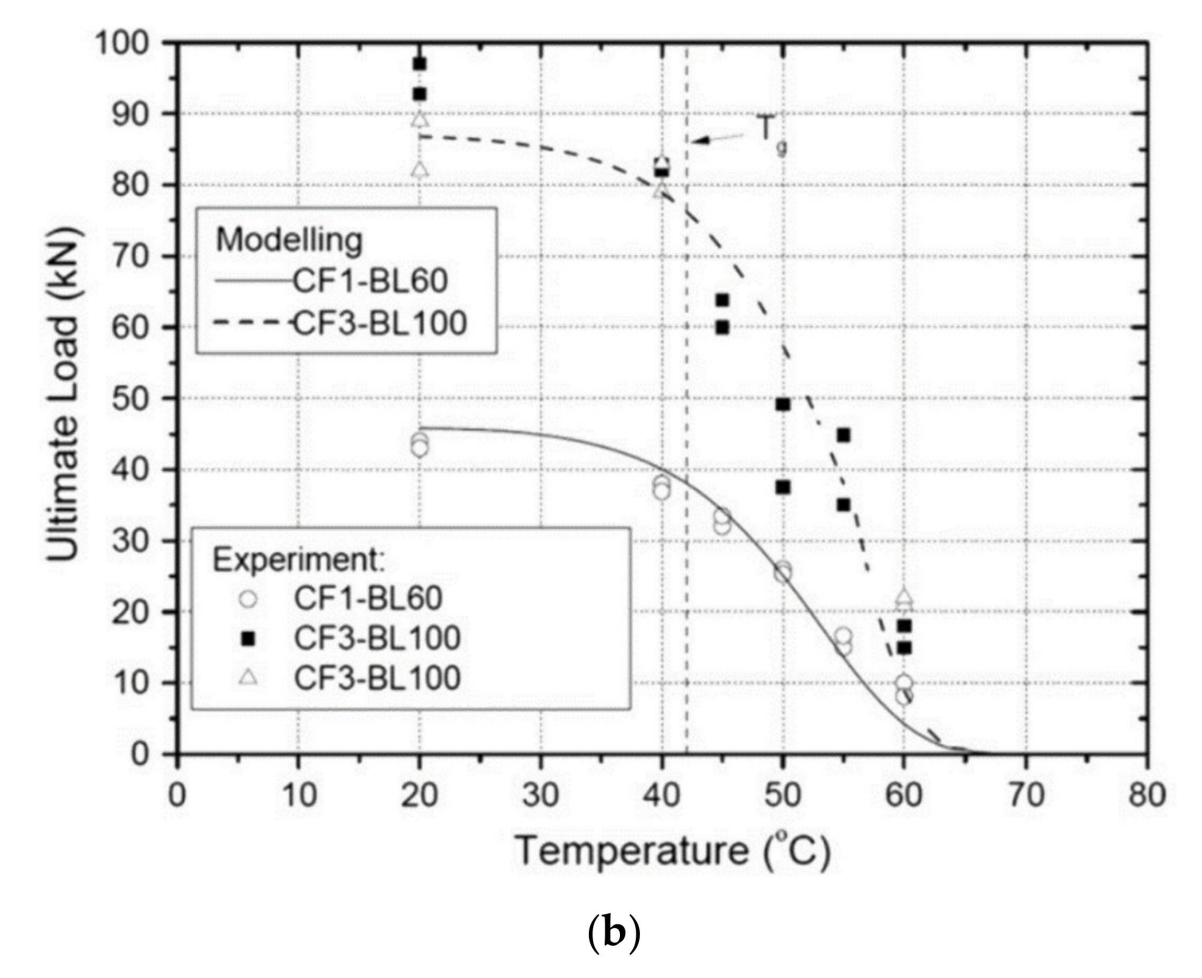
(**a**) Normalised strength (f_fu_), Young’s modulus (E_f_ chord) and ultimate strain (ε_fu_) of samples tested at 70 °C and 65% relative humidity [71]; used with permission from constructions and building materials, Elsevier, 2021. (**b**) Experimental and modelled bond strengths of normal modulus CFRP double lap joints vs, temperature [75], where the CF3-BL100 curve with the triangle marker is taken from [74]; used with permission from Composite Structures, Elsevier, 2021.

Studies on hybrid carbon fibres exposed to extreme temperatures ranging from 16 to 200 °C showed extreme reductions in tensile strength [70]. However, as temperatures exceeded the Tg tensile strengths tended to stabilise and remain constant. CFRP coupons were examined under extreme temperature and humidity by Di Ludovico et al. [71]. Coupons were prepared with neat epoxies and epoxies embedded with nano silica particles. Significant reductions in strength (approximately 20%) were witnessed in the commercial epoxy when tested at elevated temperature compared to in ambient conditions. The addition of nanoparticles allowed the reduction in strength between temperatures to remain negligible, as the embedment increased the Tg of the epoxy adhesive Figure 6a.

Nardone et al. [72] investigated the tensile properties of epoxy based CFRP composites under extreme service conditions. Conditioning varied from freeze–thaw cycles to temperatures of 70 °C. Freeze thaw cycles proved to not significantly influence the mechanical properties of the samples. Conversely temperatures of 70 °C caused decreases in tensile strength of approximately 30%.

Further examination into the effects of subzero temperatures on CFRP/steel joints prepared with several epoxy adhesives was conducted by Al-Shawaf [73]. It was found that both Araldite 420 and Sikadur-30 witnessed no change between sub zero and ambient temperature tests. MBrace Saturant had a significant decrease at −40 °C, due to its inferior mechanical properties and general incompatibility with steel adherends.

Temperature cycling was investigated by Nguyen et al. [61] as CFRP/steel joints were subjected to cycling temperatures between 20 and 50 °C for 1000 h at 90% relative humidity. These joints showed less than a 10% reduction in strength and stiffness after exposure, which was less than those that underwent constant 50 °C exposure for the same duration at identical humidity. These studies particularly highlight the damage associated with elevated temperatures more so than freezing, or cycling temperature exposure.

Regarding the effects of high temperatures on CFRP/steel systems, Al-Shawaf et al. [74] found that the failure modes of all CFRP configurations changed to debonding when temperatures extend beyond the adhesive’s Tg. However, if the exposure temperature is close to the glass transition temperature of an epoxy adhesive, mixed modal failure may occur. Under ambient exposures he concluded that lap joints consisting of Araldite 420A/B maintained the highest average capacities. Compared to the ultimate strength results by Nguyen et al. [75], the graphical summary (Figure 6b) shows that strengths dramatically reduce after exposure temperatures reach the epoxy’s Tg. More importantly, the decrease accurately correlates to the stiffness losses of the adhesive alone at these temperatures, proving how, at elevated temperatures, the epoxy adhesive becomes the weakest link in the system. This is further proved by the failure mode transition from CFRP delamination at ambient temperature to cohesive failure and steel-adhesive interfacial failure at temperature of 40 and 60 °C.

Similar specimen configurations and temperature exposures were investigated by Liu et al. [76], with the use of HM CFRP instead of NM CFRP. From this comparison, it appears that the modulus of the CFRP plays a significant role in the failure mode of tests conducted at elevated temperatures. With double lap joints constructed with HM CFRP witnessing CFRP rupture in both ambient and elevated temperature conditions.

It appears that strength variations rely heavily on the CFRP modulus, exposure temperature and hence the Tg of the epoxy used for CFRP adhesion. The Tg can significantly alter the load transfer capabilities, as well as the failure mode of the system, which if varied can result in dramatic reductions in strength. Despite the issues with temperature, the UV radiation associated with these environments has very little effect on bond strength and failure modes [63].

The behaviour of CFRP/steel joints at these elevated temperatures is lacking clarity, with very few studies focussing on temperature effects combined with moisture or dynamic loading scenarios.

#### 2.3.6. Moisture and Saturation

Steel structures requiring retrofitting are often located close to seawater, and the detrimental effect of sea water on the adhesive bond of FRP/steel systems is relatively unknown. Problems with the durability of adhesively bonded CFRP joints may occur if the epoxy adhesive or CFRP matrix suffer cohesive losses from the plasticisation by water [77]. Despite predominately experiencing losses in bond strength, occasionally configurations can show evidence of insensitivity to a moist environment [64,78,79]. This uncertainty has prompted examinations into the joint strength and behaviour of CFRP/steel joints exposed to moisture and saturation.

Early studies into adhered, butt jointed, mild steel substrates, such as the one conducted by Gledhill and Kinloch [80], gave insightful discoveries into environmental impacts on the composite adhesion field. In such studies, water immersion considerably reduced the strength of the steel joints. Furthermore, as temperatures exceeded the Tg of the adhesive, water migration becomes greatly accelerated. In addition, as the loss of mechanical properties occurs at elevated temperatures, which may be above the outdoor environment, these accelerated exposures may not exactly replicate the expected climate and hence may not accurately reflect environmental exposure.

Abanilla et al. [81] made some preliminary findings into the effects of moisture absorption and performance of wet layup carbon fibres in epoxy resins. The specimens were prepared at room temperature and exposed to several solutions including deionised water, 5% NaCl solution and an alkali solution. Moisture uptake showed to reduce the glass transition temperature of the epoxies used, which can have dramatic influence on bond performance, especially if elevated temperatures are expected. Most reductions were found to be resin-based and hence are at least partially recoverable, particularly those experienced during short term exposures.

The effects of distilled water on the tensile and fatigue properties of carbon fibre composites prepared with three separate adhesive matrices were investigated by Selzer and Friedrich [82]. Distilled water submergences caused no change to the mechanical properties of the composites, regardless of the temperature (all below Tg) or duration of exposure (beyond specimen saturation). These findings support the importance of the temperature of exposure in relation to the Tg of the epoxy matrix or adhesive and its moisture uptake or absorptivity.

Other studies have examined the degradation caused by the combination of humidity and temperature. One study into the bond durability of carbon fibre/epoxy composite joints exposed specimens to 90% relative humidity at 40 °C for 12 months [83]. Results identified a steady decline in bond strength of the joints tested after various exposure periods. Another study conducted wedge tests on specimens patched with GFRP and CFRP sheets after a variety of environmental exposures [1]. Results showed that “hot” water (65 °C) was the most degrading condition followed by sea and ambient waters. Freezing conditions and freeze thaw cycles remained the least influential and overall a recommendation of a hybrid carbon and glass fibre composite system was recommended. The glass fibres, placed closest to the steel surface, would help prevent the likelihood of galvanic corrosion as well as providing extended durability.

**Figure 7:**
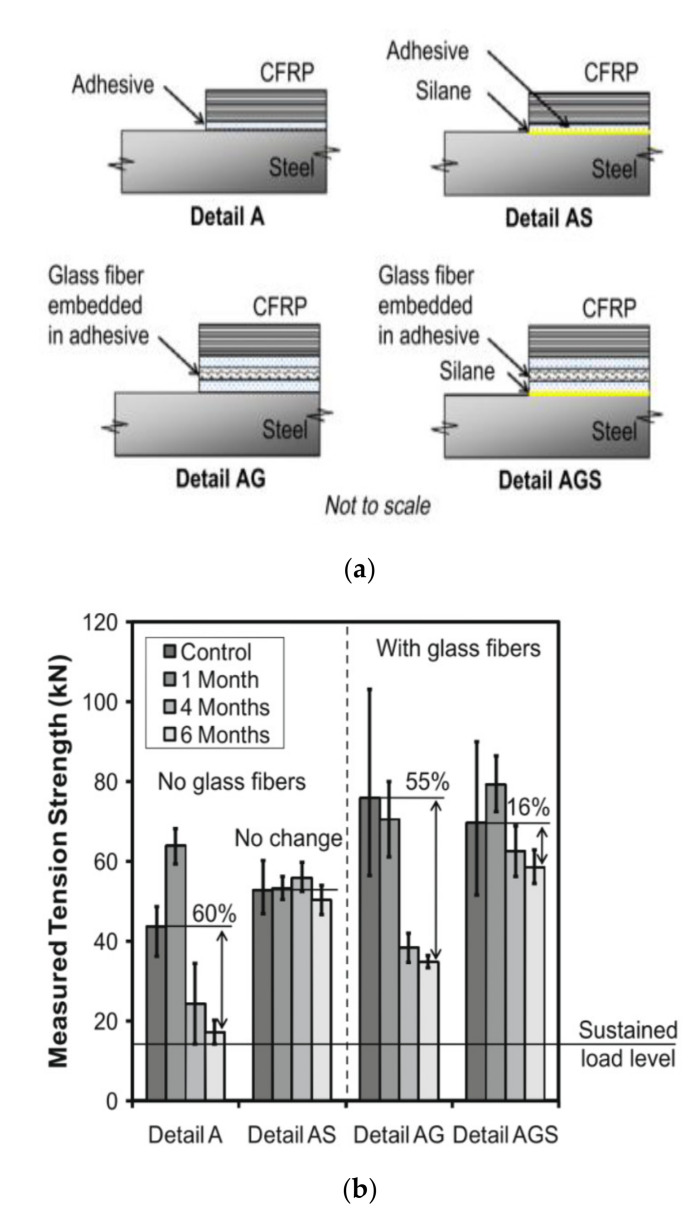
(**a**) Schematic representations of specimen configurations used in double lap bond joints. (**b**) Joint strength of various configurations after exposure with sustained load [59]; used with permission from Construction and Building Materials, Elsevier, 2021.

With this in consideration a recent study utilised various configurations of FRP patches (Figure 7a) and primers, as preventative measures, to determine the most suitable application for synthetic sea water [59]. The pre-tensioned double lap joints were submerged for up to 6 months before being static tensile tested until failure. Results indicated that the use of a glass fibre layer increased the ultimate strength of the specimens, while the silane primer showed great ability to improve the durability of the specimens during longer exposure durations (Figure 7b). Failure was likely due to the deterioration of the interfacial bond of the steel and the adhesive as well as the deterioration of the adhesive itself. Silane creates a stronger chemical bond between the steel and the adhesive, hence the increased durability.

Further studies have investigated the effects of a combination of seawater, temperature and humidity on tensile behaviour of CFRP/steel strap joints [62]. Dissimilar to the previous study these specimens were unloaded during exposures. Results showed a 9% and 16% further loss in the strength and stiffness, respectively, between the 20 °C and 50 °C exposures. Strength and stiffness degradation increased with higher exposure temperatures, this rate was reportedly faster in the initial stages than after prolonged exposures. Again, the degradation experienced in the lap joints was similar to that of the adhesive coupons, under similar exposure, hinting that degradation is caused by the adhesive itself. Conversely, specimens at 90% relative humidity and 50 °C exposure experienced only a 10% decrease in strength after 1000 h. Importantly, despite exposed and unexposed specimens mostly delaminating, no corrosion deposits were witnessed on the steel’s surface when small regions of cohesion failure occurred.

The effect of temperature-controlled seawater saturation was investigated by Agarwal et al. [60] on CFRP/steel single lap joints. It was found that when the saturated temperature remained around or below the Tg of the patching adhesive, bond strengths remained at least 85% of the ultimate bond strengths. Although, it was noticed that despite the relatively small reduction in strength the failure mode varied under certain temperature ranges. This highlights the sensitivity to wet thermal cycles, which may be more significant depending on the change in failure mode. It has been stated that adhesive fatigue resistance experiences reductions when exposed to water, although more testing is still required under combined moisture and thermal effects [79].

Seica and Packer [84] examined patching of tubular structures, primarily for wet or underwater applications. The use of tubular members come with inherent connection fatigue issues and often require strengthening. This investigation focused on the rehabilitation of members associated with the offshore industry and underwater repair. Even with underwater repair methods, all composite members resulted in improved structural performance. Importantly the specimen which utilised a lighter more manipulative fibre and a resin specifically designed to cure underwater performed comparatively well compared to those cured in air.

Thus, not only have the moisture effects of CFRP/steel bond durability been primarily studied in isolation from other physical conditions such as fatigue loading. Consequently, the combined effects of fatigue loading and environmental conditioning on adhesively bonded steel/CFRP joints remains a critical requirement [85]; primarily because when these conditions are combined, they can cause significant reductions in strength and stiffness [2].

## 3. Current Remedies for Bond Degradation

Several remedies have been proposed to minimise and potentially remove the degradation issues that have been discussed in Section 2. The following sections outline new and innovative techniques for durability improvement of CFRP/steel joints, beyond curing techniques and adhesive selection that were previously discussed.

### 3.1. Material Insulation

In order to prevent the galvanic interaction, or to improve the bond durability and strength of CFRP/steel joints, material layers have been employed to insulate and isolate the two adherends. Firstly, it is recognised that epoxy adhesives are considered insulators and can, for the most part, prevent the interaction of CFRP with steel. However, if the adhesive layer is damaged, degraded or is not prepared sufficiently, contact is still possible. Along with adhesives, layers of GFRP (non-conductive) have thought to be an appropriate insulator between CFRP and steel materials to reduce the likelihood of galvanic interaction [86] and hence improve durability. This process however has been met with mixed results.

Early applications showed that GFRP layers exhibited comparable corrosion resistance to chemical solvents in salt-spray environments [87]. However, the addition of a GFRP layer is often described as being less durable than the adhesive alone [32]. This decreased durability is associated with the wicking properties of the GFRP material, especially as a fabric or sheet, which causes water ingress to increase more than adhesive itself [88]. This moisture absorbance may lead to an increase rate of adhesive degradation and hence decreased strength. It must also be noted that rarely do materials retain their insulating properties after a few years due to chemical attack, wear or electrolyte absorption [89].

On the other hand, incorporating a GFRP layer has shown to enhance the bond strength of joints [90,91] by allowing a more gradual stress transfer between CFRP and steel [92]. Photiou et al. [48] achieved a 26% increase in joint strength with the inclusion of a GFRP layer over those solely configured with CFRP. Conversely those exposed to elevated temperatures achieved similar bond strength to configurations involving only CFRP [70]. In another study a GFRP layer increased initial bond strengths by more than 70% [59]. However, when specimens were submerged in 38 °C seawater for 6 months they attained comparable reductions in strength, implying an absence of resilience. In this investigation the best strength retention was achieved with surface priming rather than CFRP inclusion.

It appears from these studies that GFRP insertion is advantageous for air or room temperature applications; however, GFRP may be detrimental for systems involving environmental exposure.

### 3.2. Surface Priming

Surface priming is designed to achieve primary chemical bonds between the organic polymers and the inorganic metallic surface, which do not commonly form without pre-treatment [93]. A common priming agent with the use of epoxy adhesives and steel are organosilanes. They are commonly used for corrosion protection, but certain functionalisation (alkoxy–silanes) can also be utilised for enhanced adhesion [94]. A detailed review of the corrosion protection properties of organofunctional silanes identified the universal applicability of the primer, as well as the potential of creating hydrogen and covalent bonds with metals [95].

From this, silane coupling agents have been applied and researched in their use with adhered CFRP and steel joints. Primarily the hope of silane pre-treatment is to produce strong and durable bonds, importantly in the presence of water and water vapour [96]. γ-GPS (glycidoxypropylmethoxysilane) solutions in particular have shown great ability to improve the levels of durability of grit-blasted specimens over other surface preparation techniques [97]. However, it must be noted that a silanes applicability is heavily affected by its compatibility with the adhesion polymer and their accompanying functional groups.

A detailed investigation by Walker [98] explored the use of silane as an additive to epoxide paints and as a steel surface pre-treatment. Pre-treatment caused all steel samples to achieve higher bond strengths than those that were untreated. This was primarily caused by a reduction in the coating area detachment associated with silane priming. The addition of silane into the epoxy paint showed comparable results as detachment areas reduced with silane inclusion.

**Figure 8:**
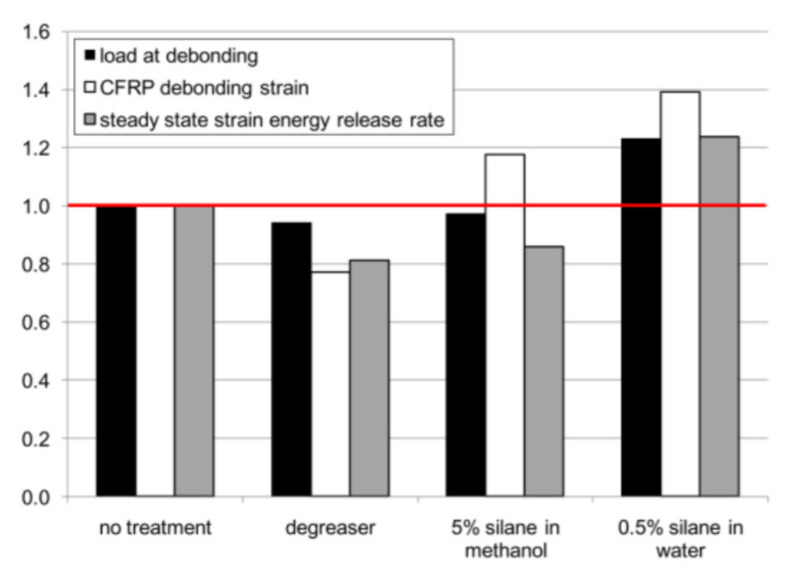
Effect of treatments normalized to behaviour of specimens having no treatment [50]; used with permission from Composite Structures, Elsevier, 2021.

Sizemore et al. [50] conducted 4-point bending tests on CFRP/steel members in order to investigate whether silane treatment improves bond performance or it simply enhances the bond performance by preventing corrosion of the steel surface during preparation. Specimens were left for 22 h between surface preparation and FRP application. Four configurations were prepared with their normalised results shown in Figure 8. Most considerably is the increase of all parameters when silane was prepared with water rather than a higher concentration in methanol. This is because the water-based solution creates the necessary environment for silane to hydrolyse and immediately form the polysiloxane film. Methonal, on the other hand, does not provide this essential setting and may also cause silane to evaporate with it while standing or drying.

Research into the preparation of silane primers used for adhesion promotion also indicate that the pH of the solution can significantly alter the bond effectiveness. Tod et al. [99] found that an acidic solution produced a 40% increase in strength compared to the untreated sample, while the alkali version decreased by 40%. This study also showed that silane concentration and drying conditions can vary the lap shear strength by approximately 30% and 20%, respectively. Gledhill et al. [100] confirmed the importance of pH and drying conditions in regards to the moisture resistance of silane films, but went further in describing how each organosilane system would have their own set of optimum conditions.

Accelerated environmental testing was conducted by Knox and Cowling [101] on epoxy adhered steel lap shear specimens. The effect of silane pre-treatment was examined by comparing the original dry strength to the residual strength after aging of configurations with and without silane. Silane primers produced the best results under the extreme conditions of 30 °C and 100% humidity confirming their potential as a suitable primer for mild steel/epoxy joints.

The long-term durability of silane pre-treated CFRP/steel joints under seawater exposure was investigated by [59]. In which, the application of silane, as the sole joint treatment, created complete joint strength retention even after 6 months submergence at almost 40 °C. The configuration that also included a glass fibre insulation layer obtained a higher initial strength but was far more susceptible to water degradation, witnessing a 55% reduction over 6 months. These findings denote the ability of silane to improve durability of CFRP/steel joints, which outperform the use of GFRP insulation layers.

Recently, Borrie et al. [102] investigated the use of silane chemical barriers and adhesive modifications to improve bond durability and retard environmental damage and restrict bond degradation. They found that silane coatings improved in strength and durability during environmental submergence, with a 7% and 17% improvement in strength of Araldite 420 HM CFRP joints after submergence at 20 °C and 50 °C, respectively. This is likely due to the increased hydrolysis though water exposure, slowing the rate of degradation. Silane is best suited when failure modes are found to be in the interface between the steel and adhesive, such as laminates multilayered CFRPs and joints under moisture attack [102].

Chemical priming of the steel surface has shown to be very effective in increasing the joint strength retention under extreme environmental exposure. Most significantly, the requirement of hydrolysis gives it a heightened relevance for applications involving moisture exposures or submergence.

### 3.3. Adhesive Modification

The physical and mechanical properties of adhesives can be altered through various processes. One process which has recently been investigated involves the embedment of nanoparticles into the epoxy adhesive. One current option involves the use of carbon nanotubes (CNT). These particles act as reinforcement to the adhesive, varying mechanical strength and modulus as well as the Tg of the adhesive.

Improvements from CNT embedment rely on the adhesion with the resin, the distribution of CNT particles and the aspect ratio of the tubes [103]. CNT’s modulus properties can reach upwards of 1 TPa and a strength greater than high strength carbon fibres, all for a fraction of the weight of steel. CNTs have shown incredible mechanical properties as well as impressive ability in reinforcing polymer-based materials [104,105,106,107].

Ideally a lower viscosity adhesive is suited to CNT embedment as it allows a more uniform dispersion of the tubes [108]. Furthermore, one key consideration is that CNT dispersion can often cause an increase in adhesive viscosity [109], making it less workable in structural applications. This workability may also affect the saturation of fibres when wet lay-up techniques are used with CFRP sheeting materials. Beyond this, Puglia et al. [109] also found that the thermal conductivity of the CNTs also caused the adhesives to cure at a faster rate.

**Figure 9:**
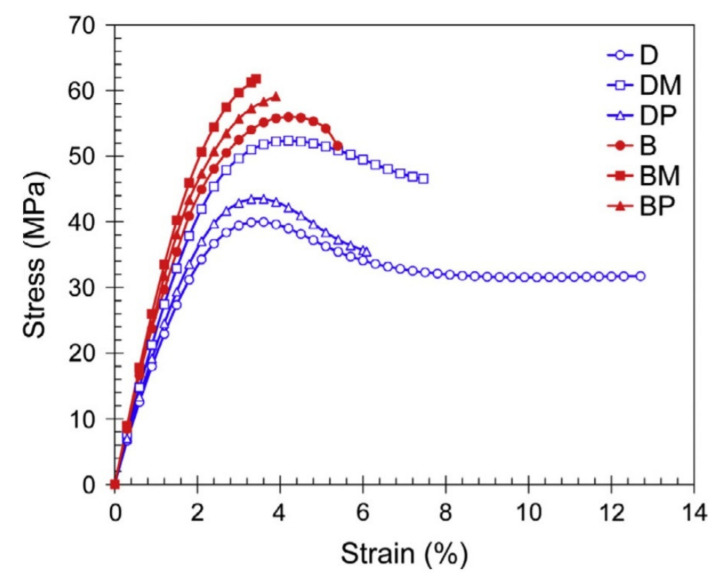
Tensile strain–stress curve of pure epoxies and their corresponding CNT reinforced composites. D: ductile epoxy, B: brittle epoxy, P: CNT powder and M: CNT masterbatch. The tensile test results are the mean of three experiments with a standard deviation around ±2%. [111]; used with permission from Composites: Part A, Elsevier, 2021.

Recently, Korayem and his team conducted several investigations into the CNT modification of structural adhesives. A 3 wt.% CNT inclusion to a ductile epoxy adhesive resulted in a 20% increase in elastic modulus, 30% increase in tensile strength and a 21.1 °C increase in the Tg of the adhesive [110] (Figure 9). Furthermore, these modified adhesives were used for bonding CFRP to steel. The performance of the bonds was adjudicated through pull off tests [111] and double strap joints [107]. The pull off tests were conducted at temperatures up to 70 °C and produced strengths twice as high as those fabricated with the neat epoxy adhesives.

In the lap joint tests, specimens with short bond lengths witnessed small increases in bond strength after the inclusion of CNTs. Conversely, larger bond lengths (120 mm) resulted in a 5% reduction in strength with the addition of CNTs. These small changes were because of the unchanging failure modes of both configurations. As the weakest element in both systems was the interface between the steel and epoxy adhesive, the improvement of the CNT embedment was concealed.

Along with surface priming, it appears that epoxy modification is a viable method to improve the strength of CFRP/steel joints, especially under temperature exposure. However modified adhesives have not been used in conjunction with surface primers or under environmental exposures.

## 4. Behaviour of CFRP Repaired Steel

Corrosion damaged and fatigue loaded steel materials witness high stress regions which can often result in significant crack formation. If not found or left unrepaired they may continue to propagate and eventually cause failure. Older methods of repair welding, metal reinforcement and crack arrest holes are becoming obsolete, potentially being overthrown by the use of stiffer FRP materials to reduce crack propagation rates and regain structural integrity.

It is important to recognise that the damages and remedies that affect joint performance of CFRP/steel systems outlined in Section 2.3 and Section 3 are entirely applicable to those used for patching or repair of pre-damaged steels. However, this area is far less researched than bond durability and hence its performance if severely unknown. The following sections will outline the limited studies on the topic of CFRP repaired steel and their durability to environmental exposure.

### 4.1. Experimental Repair of Fatigued Steel Plates

Structural repair using FRP materials developed through the patching of aircraft, which primarily are constructed with aluminium alloys. As such several investigations have researched the fatigue performance of FRP repaired aluminium [112,113,114,115,116]. However due to the comparable stiffness to steel, CFRP has become the dominant rehabilitation material for the repair of steel structures.

**Figure 10:**
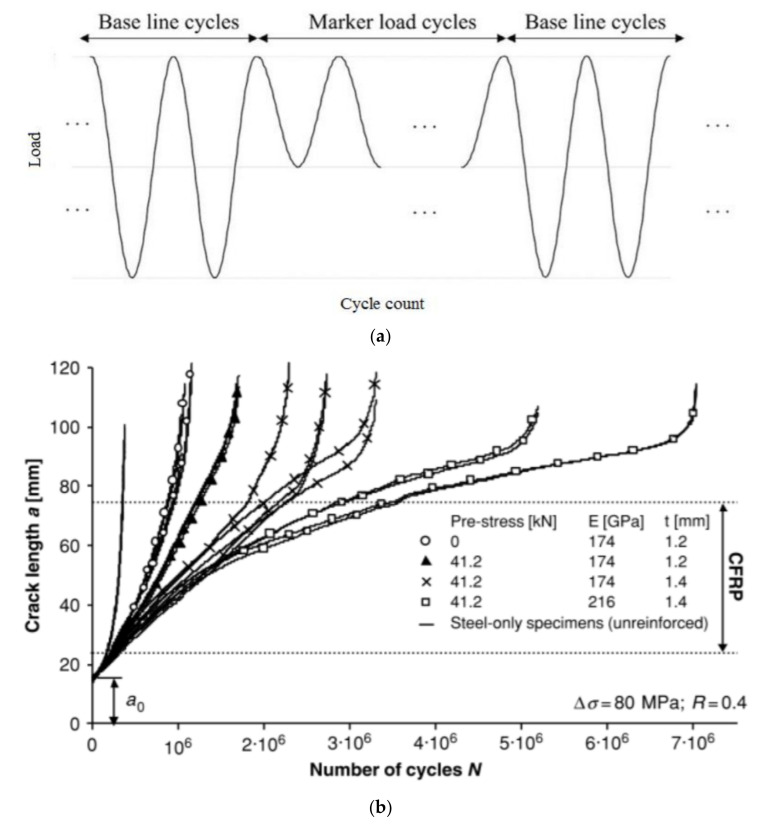
(**a**) Schematic representation of “beach marking” cycles adapted from [120], with permission from Thin-Walled Structures, Elsevier, 2021. (**b**) Crack propagation curves of tests conducted at the SFIT, published in [121]; used with permission from Fatigue & Fracture of Engineering Materials & Structures, John Wiley & Sons Ltd., 2021.

Majority of these studies steel plates utilise the “beach marking” technique to investigate and quantify the crack propagation. This involves varying the stress range applied to the sample for a pre-set number of cycles (Figure 10a). This technique creates visible marks left on the fracture surface on the material during load amplitude variations. When the load amplitude is changed, the stress intensity at the crack tip is altered and hence the surface undergoes a different fracture rate. This varying fracture rate results in a distinctive colour change on the fracture surface which can be treated as a time stamp in relation to your known applied cycle sequence. This technique is widely used and accepted as a good method to retrieve crack propagation information which is otherwise hidden below FRP reinforcement.

An extensive experimental and numerical study was conducted at the Swiss Federal Institute of Technology (SFIT) where they investigated CFRP fatigue repair of full-scale riveted bridge beams [117] and small-scale steel plates [118,119]. Fatigue cracks permeated from the rivet holes and were considered likely to propagate and cause failure. Initially, small scale steel plates with transverse cracks were manufactured to investigate the potential of CFRP repair. Under a stress ratio of 0.4 the non-prestressed CFRP repaired plates increased their fatigue life by a factor of three. Once prestressing of the CFRP laminate to 41.2 kN was introduced, the fatigue performance doubled. Furthermore, when a CFRP laminate that was 35% stiffer was also prestressed to the same level the fatigue life increased by a factor of twenty. Full crack propagation curves for these tests are displayed below (Figure 10b), highlighting the various propagation rates dictated by the loading and specimen configuration.

The full-scale riveted beams were removed from a railway bridge in Thusis, Switzerland, after its deconstruction and were repaired using CFRP laminate. Two of the 5.1-m-long girders were tested under four-point bending, constant amplitude fatigue loading, after the bottom flange was repaired with CFRP. With five layers of CFRP, of which three were prestressed, crack propagation seemed to have ceased.

Okura et al. [122] investigated the use of CFRP sheeting materials to improve the fatigue performance of pre-cracked steel plates. Specimens were prepared with either one or two initial holes and were patched with CFRP sheeting with an elastic modulus of 437.3 GPa. Single hole specimens repaired with two layers of CFRP sheet witnessed improvements of 15 times, which failed to increase even when the number of layers was increased to ten. Those with two holes and two layers of CFRP improved by a factor of 33 times and similarly to one hole did not improve when the number of applied layers increased.

The fatigue performance of CFRP repaired central and edge notched steel plates were investigated by Jones and Civjan [123]. Constant amplitude fatigue loading was applied at 25 Hz until complete through failure of the plate occurred. Several parameters such as the bond length, repair before or after crack propagation and single- or double-sided repairs were investigated. When patching was applied before natural crack propagation the CFRP successful delayed the formation and extension of fatigue cracking. It was also shown that applying the CFRP directly over the crack, or its assumed path, was preferred to prevent premature debonding. Additionally, to prevent debonding it was highlighted that surface preparation and adhesive performance were crucial in repair performance.

Further results on edge notched specimens were published by Colombi et al. [124], who cited sudden failure between the adhesive and steel interface once crack lengths reached approximately 70–80% of the plates width. Noticeably repair methods were more efficient when initial crack lengths were minimised, i.e., patching repairs were conducted as early into propagation as possible. Finally, similarly to previous studies it was noted that the most effective patch configuration was when the entire steel plate was covered by the reinforcement.

Zheng et al. [125] studied the effects of CFRP modulus, single- and double-sided patching as well as fatigue stress ranges on repaired steel plates. It was found that fatigue lives improved by a factor of 1.55 to 5.8 depending on the configuration and loading. Specimens with the lower (60–150 kN) fatigue loading, higher modulus CFRP and double-sided repair outperformed all others.

The use of stop holes in conjunction with partial and full CFRP repair was experimentally investigated by Suzuki et al. [126]. During this study, electrical gauges were utilised to accurately record crack initiation and propagation rates from the edge of the stop holes. It was found that the combination of stop holes and CFRP performed better than either of them separately. The longest fatigue lives were achieved when stop holes were used in conjunction with patching the entire steels width with CFRP.

**Figure 11:**
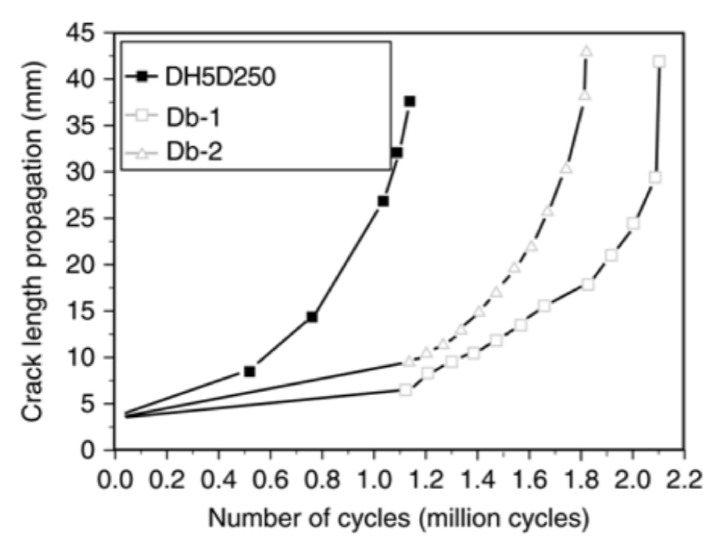
Fatigue life curve of specimens with a completely covered initial crack with normal modulus CFRP [128], with permission from 2021 Composite Structures, Elsevier, UHM CFRP [54], with permission from Advances in Structural Engineering, SAGE, 2021.

One such study [127] involved patching notched steel plates with an initial crack length of 60 mm using carbon fabrics. Fatigue life of patched specimens was extended by up to twice as long as unpatched specimens. It was noted that specimens having unsymmetrical, single sided patching, experienced out of plane bending issues during loading. When thicker stiffer patches were used eccentricity of the tensile forces increased and hence bending forces increased causing early debonding in samples. Further studies [128] showed that one sided patching caused non-symmetrical crack propagation rates through the steel’s surface. The patched side of the crack has a slower rate of propagation due to the stiffness increase from the CFRP patching. High modulus sheeting increased fatigue life by 4.7–7.9 times, while normal modulus sheeting increased by up to 2.7 times. In another study, Wu et al. [54] used ultra-high modulus (UHM) CFRP laminates to replicate configurations of past crack growth studies for comparison. They found that UHM laminates were more advantageous in improving fatigue lives when compared to that of normal modulus laminates (Figure 11). With extensions of up to 7.47 times the reinforcement using UHM laminates was comparable to prestressed CFRP materials.

Yu et al. [129] recently experimented on the effect of CFRP repair for different damage levels (crack lengths). The damage levels represented cracks found at various stages in their formation and growth. With damages ranging from 2% to 20% of the steel plates width CFRP laminates were applied either side of the initial crack front. Increasing the damage degree resulted in significantly shorter fatigue lives with the 20% damaged specimen surviving less than 30% as long than that of 2% damage. However, strengthening of the 20% pre-damaged specimen resulted in the highest fatigue life improvement of 186%.

Similar patching techniques were then applied for the repair of gusset welded joints [130]. Two separate stress ranges were tested in order to compare the effectiveness of the repairs under varieties of loading. Under both stress ranges of 120 and 150 MPa the double-sided repairs with ultra-high modulus CFRP produced the largest fatigue life increase. Specimens with single side repairs reached fatigue lives 1.44 times longer than the controls and double-sided repairs improved by 8.17 times.

CFRP retrofitting has proved its ability to reduce crack propagation rates. Ideally the entire crack length should be covered, although if that is not possible, the reinforcement should be placed most closely to the crack tip.

### 4.2. Strengthening of Steel Members under Static Bending

Along with numerous smaller scale experimental analyses, a limited number of large-scale, detailed, applications of fibre reinforced polymers have been investigated. These applications are designed to more closely replicate field applications and are generally undertaken on elements such as composite beams, steel beams and cross beams.

Al-Saidy [131] manufactured six simply supported, steel-concrete composite beams for testing under static 4-point bending. Three of the 3.4-m-long beams had a combination of CFRP plates patching their web and tensile flange, the other three beams were used as controls. Important findings included flexural stiffness increases by up to 50% after patching; strength can be fully restored to its original undamaged state. However, patching does slightly reduce the ductility of the system. Interestingly no bond issues were witnessed; this may have been due to the fact that over 90% of flanges length was covered by CFRP.

Furthermore, Schnerch and Rizkalla [132] rehabilitated three 6.4-m spanning steel-concrete composite beams, patched after an initial loading cycle. The initial loading cycle, applied using 4-point bending, was designed to induce a strain of 0.12% in the tension flange, equivalent to 60% of its yield stress. After this, the beams were patched with various types and configurations of CFRP strips, using Spabond 345 epoxy. After rehabilitation a secondary loading cycle was applied, again to 0.12% strain in the tension flange, before being loaded to failure. Results showed 10 to 34% increases in stiffness, as well as up to 46% increase in ultimate strength. The prestressed CFRP showed the most economical use of the material by increasing stiffness while maintaining comparatively normal ductility.

In a similar study, Sen et al. [133] manufactured six identical 6.1-m steel–concrete composite beams, which were strengthened using CFRP laminate materials, covering the full width of the beams tension flange. The 3.65 m long laminate was place in the beam’s midspan, before undergoing 4-point bending analysis. It was found that the strength and stiffness improvements of these beams was less than that seem for concrete and wooded structures. CFRP applications increased the strength of all specimens, ranging from 9 to 52%. Immediate failure was witnessed for one specimen prepared with adhesive bonding and 5 mm thick CFRP laminate, which initiated through adhesive failure. Thinner CFRP materials did not witness the same sudden failure as the transferred stresses never reached the shear stress of the adhesive. This large scaled testing highlighted the importance of anchorage to prevent premature debonding and sudden failures.

The effects of multi-layered CFRP strengthening was examined by Tavakkolizadeh and Saadatmanesh [134] on several steel-concrete girders. The 4.78 m girders were patched with one, three or five layers of low modulus (144 GPa) CFRP sheeting on the external surface of their lower flange. The CFRP was applied with a 150 mm offset in order to stagger the thickness reduction of the CFRP fabric layers. The ultimate loads of the girders under four-point bending improved by 44, 51 and 76% for the one, three and five layered specimens respectively. The relatively flexible adhesive meant that the stiffness of the beams did not dramatically change between configurations. As the number of layers increased, their load carrying efficiency decreased, with one-layer systems holding 75% of its ultimate strength compared to 42% in the five-layer case. Identical beams were then prepared with initial damages equal to 25, 50 and 100% losses of the tension flange before being repaired with layers of CFRP sheeting [135]. This technique of tensile flange notching was used earlier by Liu et al. [136] to successfully simulated the corrosion steel beams which showed increased stiffness and plastic load bonding CFRP laminates to these simulated corroded beams again proved the ability of CFRP to increase the stiffness of beams as well as the plastic load of the beams. It was chosen that one, three and five layers be used for the repair of 25, 50 and 100% losses, respectively. The repair configurations resulted in 20, 80 and 10% increases in ultimate load capacity for the increasing damage levels. It appears that if damages are not repaired early enough, then CFRP application does not give an effective increase in strength, however if rehabilitation is timely significant improvements can be expected. The elastic stiffnesses of the beams were 91, 102 and 86% of the intact beams, while postelastic stiffness increased dramatically to 21, 19 and 32 times the undamaged girder.

Shaat and Fam [137] investigated the repair of 1.96 m long cracked steel beams supporting concrete slabs. The cracks were designed to simulate a fatigue or corrosion damage and were patched with standard modulus and high modulus CFRP sheets. After repair and monotonic four-point bending, both CFRP repair systems recovered to the strength and stiffness of the original composite beams. High modulus sheeting created a 10 and 26% improvement over the original strength and stiffness respectively. Premature debonding issues were commonly witnessed in beams repaired with standard modulus CFRP with the curvature of the beam under loading contributing to this phenomenon. Conversely, the high modulus systems resulted in CFRP rupture due to the lower rupture strain associated with the increased modulus.

**Figure 12:**
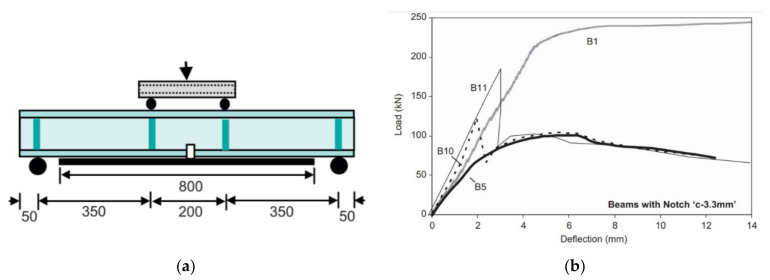
(**a**) Test set up of CFRP repaired notched composite beams. (**b**) Beams with full cut, Notches type “c” [138]; used with permission from Thin-Walled Structures, Elsevier, 2021.

Fam et al. [138] then investigated the use of CFRP materials on steel (non-composite) beams under four-point bending (Figure 12a). The removal of the entire steel flange resulted in a reduction in flexural strength and stiffness of about 60%. After CFRP repair flexural strengths increased up to 79% of the undamaged intact beams. High modulus CFRP repairs resulted in varying failure modes depending on the type and cross-sectional area of the material. Figure 12b shows there was a small increase in ultimate capacity of the repaired beam (B10) with CFRP sheets over that of the control notched beam (B5). However, the elastic stiffness was increased significantly and even exceeded that of the intact control beam.

An American study by Nozaka et al. [139] explored the use of multiple CFRP materials and structural adhesives for their effects on repairing structurally damaged (fatigued) beams. For this study, CFRP materials were applied using five different configurations on a damaged steel plate before being attached (temporarily) to the large scale girder. This allows the one girder to be used while still applying appropriate bending load to the CFRP composites. The 4.3-m-long beams were loaded under four point bending with 2 m between the loading points. Increasing the number of layers allowed the beam to reach a higher maximum moment before failure, although no configurations reached the full tensile strength of the adhered CFRP strip. Furthermore, increasing the bond length of single layer repairs above 200 mm did not achieve any increase in strength.

### 4.3. Strengthening of Steel Members under Bending Fatigue

Beyond the patching techniques used on steel plates with fatigue issues, larger reinforcements can be applied to support and strengthen the critical tension regions of members under bending. Bending fatigue is a crucial consideration for the industrial application of CFRP repair as members requiring rehabilitation, such as beams, experience intense bending loads. The following section will summarise several experimental investigations into the CFRP strengthening of large-scale beams under bending fatigue. Further information may be found in a recently published review on this topic [140].

In a comprehensive study, Miller et al. [10] experimentally tested four full-scale bridge girders removed from a bridge that spanned over a creek in Schuylkill County, Pennsylvania. Due to the surrounding environment the steel girders were heavily corroded along the length of their tension flange and web. The 6.4-m girders were rehabilitated with a single layer of full length CFRP laminate bonded to the inner and outer surfaces of the tension flange. This addition of the CFRP material caused the beams stiffness to increase between 10 and 37%. The two beams statically tested under three-point bending witnessed ultimate capacity increases of 17 and 25%. Two further girders were fatigued for 10 million cycles at a stress range of 34 MPa, which was a magnitude that was considered similar to field expectations. Throughout periodic monitoring, the strengthened girders experienced no level of debonding or losses in global stiffness, hinting at a high fatigue resistance. After confirming these benefits via modelling, a field application was conducted on a single girder for long term data on CFRP rehabilitation durability. Pre- and post-retrofit monitoring indicated that an 11.6% increase in global flexural stiffness was achieved with obvious reductions in tensile strain.

Moreover, Rizkalla et al. [141] investigated the use of adhered high modulus CFRP to steel–concrete composite beams. Initially, a thorough investigation into adhesive and CFRP selection was undertaken through small scale joints and large-scale validation on steel beams. Furthermore, they investigated the fatigue behaviour as well as optimal bond and splice configurations before proposing design guidelines for the application of CFRP materials. Results proved that yield load, ultimate capacity and elastic stiffness can all be increased with the installation of HM CFRP materials. Beams also witnessed superior performance during overloading and fatigue loading compared to unstrengthened beams. Finally, a reverse tapered plate end is recommended to increase load capacity and reduce stress concentration that may cause premature debonding.

As many structures requiring rehabilitation may not be available to be completely out of order during repair and the influence of fatigue loading during adhesive curing was investigated by Nikouka et al. [142]. The steel beams were strengthened with CFRP fibres with a modulus of 310 MPa and were subjected to fatigue loading that simulated a passing train for 48 h during curing. The interfered curing process resulted in changes to the stiffness and failure load of the strengthened beams and ultimately caused the beams failure mode to change to CFRP debonding. This was the opposite to the result seen by Nozaka et al. [139] where vibrations during curing of repaired beams showed no significant variation is strength.

Suzuki [143] conducted both static and fatigue three-point bending tests on both unstrengthened and strengthened steel beams. Strengthened configurations were either patched with a traditional steel plate or with one layer of CFRP. The CFRP had a comparable elastic modulus to the steel (200 GPa) and, as such, both caused similar improvements over the unstrengthened beams. This similarity led to the conclusion that the design theory for CFRP strengthened steel beams was the same as ordinary steel beams as long as the CFRP and adhesive thickness was included in the beam’s height. The lack of premature debonding during static loading implied the adhesive remained intact throughout testing. Beyond this fatigue bending tests were conducted on beams strengthened with CFRP. Beams were strengthened with one or six layers of CFRP where the six layered beams reduced to four and two layers from the centre to the supports. Applied loading created 70 MPa of stress in the patched flange but between 10 and 50 million cycles there was no change in the stress distribution meaning that efficient fatigue strength and endurance was provided.

The effectiveness of CFRP laminate retrofitting of notched steel beams was experimentally tested by Tavakkolizadeh and Saadatmanesh [144]. 21 specimens were tested at a variety of stress ranges under 4-point bending between 5 and 10 Hz. A number of important trends were identified including: an increased fatigue life of 2.5–3.4 times larger for retrofitted beams (Table 2), larger crack lengths allowed before stiffness decreases, a 65% decrease in crack growth rates when retrofitted and 3.5 times more cycles witnessed after crack initiation to failure. This scaled experiment proved the effectiveness of CFRP laminates in resisting fatigue cycles and gave a further insight into the potential of large scaled industry applications.

The backface-strain technique was investigated by Deng and Lee [145] in order to detect crack initiation and track the deterioration of the adhesive layer of CFRP-retrofitted steel beams. Small-scale steel beams (1.2 m) were used of which nine were tested under static loads and eight tested under fatigue. It was found that although the stiffness of the retrofitted beams decreased as crack growth increased, it was considered negligible because of the thin thickness of the adhered CFRP plates. The developed S-N curve also relates the maximum interfacial stress at the plate end to the number of cycles for crack initiation, with the maximum fatigue limit being 30% of the static ultimate strength. Furthermore, they found major influences on fatigue life caused by the fatigue range and the maximum load in the load range.

Wu et al. [52] used both CFRP and steel wire BFRP polymers to retrofit steel beams with initial mid-span notches in the tension flanges. Five separate configurations were investigated, with the final stage of FRP implementation being the affixing of anchorage to the FRP systems. The 2.8-m-long beams were fatigued under four point bending with the loading points 500 mm apart at a rate of 4 Hz. The maximum load being 200 kN and the minimum load being 40 kN which represent 40% of the yield load and an estimated value of combined dead and live load, respectively. In turn, the fatigue life of the steel beams was successfully increased through FRP application by 3.33–5.26 times over the unstrengthened beam, whereas strengthening through steel plate welding only increased the fatigue life by 1.74 times. FRPs effectively prolonged crack initiation, reduced the crack growth rate, reduced residual deflection, increased stiffness retention and improved the failure mode over the weld repaired specimens. Overall CFRP prepared beams had the best strengthening effects on the fatigued beams.

**Figure 13:**
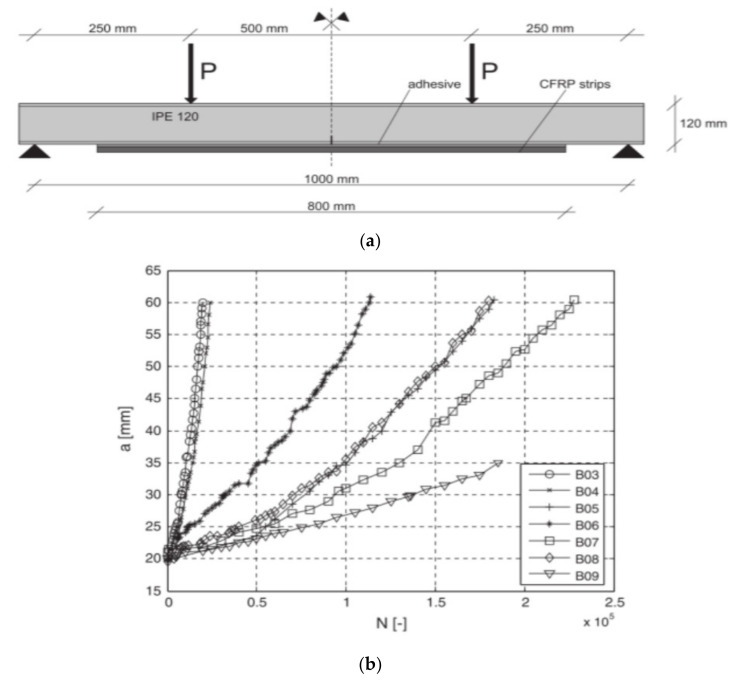
(**a**) Specimen and retrofitting geometry of repaired steel beams [148]; used with permission from Engineering Fracture Mechanics, Elsevier, 2021. (**b**) Crack propagation curves for specimens repaired with one layer (B03, B04) and two layers (B05 to B09) [148]; used with permission from Engineering Fracture Mechanics, Elsevier, 2021.

A recent European study extensively investigated the use un-bonded CFRP systems, utilising the nonessential surface preparation to reduce installation time. Various fibre moduli [31], pre-stressing [29] and fatigue resistance [146,147] were investigated for application to steel beams. These studies culminated in system fatigue strengthening of a 120-year-old rail bridge in Switzerland using their unbonded methodology [30]. In another study, Colombi and Fava [148] experimentally and analytically investigated CFRP repair of steel beams (Figure 13a) prepared with a transverse crack through the flange and partially into the web. Before CFRP application the beams were subjected to fatigue loaded until a natural crack (20 mm) was developed, failure of the beam was considered to be when the crack length reached 60 mm. Specimen B03 and B04 were patched with one layer of CFRP while the B05 to B09 were retrofitted with two layers. The application of CFRP significantly improved the fatigue life of the beams as seen in the crack propagation curves in Figure 13b, with the application of two layers providing a nine times greater fatigue life than those with one layer. This is due to the increased load taken by the CFRP instead of the steel, reducing the stress in the crack tip. Similar to plate tests, the fastest crack propagation rates were witnessed as the specimens neared complete failure. Finally, it was noted that the behaviour of FRP adhesion is governed by complex phenomenon and, as such, experimental results often display large ranges of scatter, highlighted by the dissimilar propagation curves for like specimens (Figure 13b).

Additionally, with welding’s susceptibility to fatigue cracking, CFRP wrapping was conducted to retrofit welded steel crossbeams [51]. The SHS-SHS and RHS-RHS crossbeams were fatigued until certain, predetermined crack lengths were created. After this, stop holes were drilled and multiple layers of high and low modulus sheeting were applied on several surfaces as load bearing and anchorage. This circumferentially wrapped anchorage prevented any premature debonding. Fatigue life was significantly improved although due to the several exposed, hard to secure corners, the stiffness of the system was lost rather quickly.

### 4.4. Experimental Repair with Environmental Exposure

**Figure 14:**
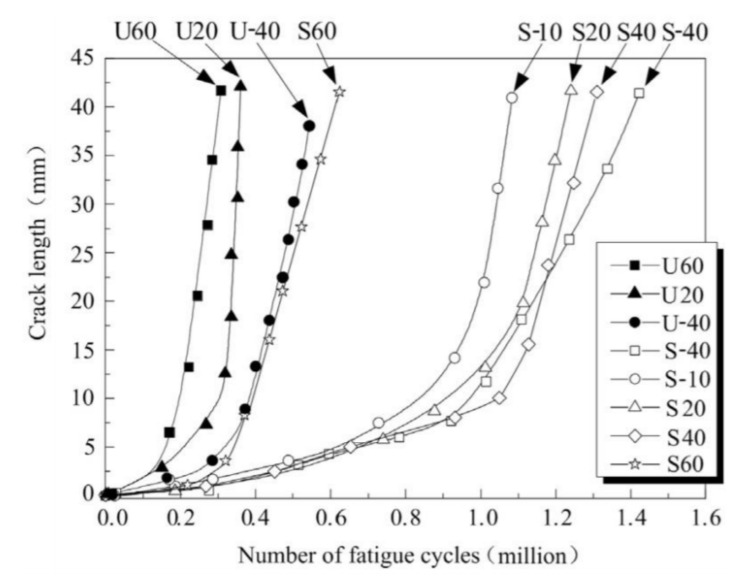
Crack propagation curves of unstrengthened, “U”, steel plates and strengthened steel plates, “S”, under temperature exposure [120]; used with permission from Thin-Walled Structures, Elsevier, 2021.

With durability issues for CFRP/steel joints discussed, knowledge in their ability to provide strengthening and crack retardation under environmental exposure is significantly lacking. One of the few studies was conducted on repaired steel plates exposed to elevated temperatures [120]. Specimens were tested in unstrengthened and CFRP strengthened (two layers) configurations. Testing was completed at temperatures ranging from −40 °C to 60 °C. Results at ambient temperature increased fatigue life by 3.4 times while at 60 °C improvements ratios dropped to 2 (Figure 14). Significantly the influence of sub-zero exposure was found to be less pronounced than elevated temperatures. This study further highlighted the poor performance of patches when exposure temperatures increase beyond the Tg of the adhesive.

To the best of the authors knowledge there are no studies to date that have investigated the crack growth and fatigue behaviour of CFRP repaired damaged steel under seawater submergence. Without this important area of research investigated the reliability of CFRP as a rehabilitation material for steel structures is substantially unknown. These studies have highlighted, but only touched on, the abilities of large scale and industrial implementations of CFRP. The sparsity of tests on steel only structures is due to the underlying unknown performances of adhered CFRP under expected industrial conditions. Until the longevity and consistent improvement of CFRP materials bonded to steel are as well established as applications with concrete, industrial implementation to structural steels will remain limited.

## 5. Prospects

In highlighting the current state of civil infrastructure around the world, previous research has exposed a necessity to update existing rehabilitation techniques. Steel structures commonly experience both extreme environmental exposure and fatigue loading, conditions that current restoration methods do not combat. Most recently, investigations into FRP materials have shown great success in improving the performance of rehabilitated structural elements. Despite significant research completed in the field of composite reinforced steel several areas remain heavily under researched. This critical review highlighted the lack of understanding and unknown resilience of these systems under industrial service conditions. In summation, the following fields of study are suggested for investigation in the future:

- Several steel/CFRP configurations successfully survive fatigue loading and environmental exposures, when applied consecutively. However, it may be more accurate and relevant to investigate the simultaneous application of environmental exposure with fatigue loading. Concurrent conditioning removes the potential for systems to experience levels of recovery once isolated from the harsh environment. Simultaneous exposure or wet/dry cycling, combined with fatigue loading would better replicate the potential extreme industrial scenarios. However, this process may create an unrealistically short exposure time, therefore, specimens may need to undergo pre-exposure to reach saturation before simultaneous loading.
- As high modulus materials exhibit superior fatigue performance, investigations into ultra-high modulus and pre-stressed CFRP laminates under environmental exposure may be valuable. Currently, more research is required to determine if the laminated materials are better at resisting environmental degradation while maintaining good fatigue resistance.
- Adhesive performance remains critical to the strength and durability of wet layup composite systems. The common epoxy adhesives used for wet layup fabrication degrade during elevated temperature seawater exposure. Hence, it may be beneficial to investigate the performance of techniques that minimise the quantity of applied adhesives, such as laminates or unbonded CFRP systems, under environmental exposure. The challenge with unbonded systems, is to find an anchorage system that maintains strength during submergence without environmental degradation.
- Real time structural health monitoring, primarily of adhered CFRP systems. With adhesive and CFRP layers preventing inspection of repaired surfaces, it would be beneficial to investigate techniques to accurately view and monitor surfaces underneath composite patches.
- Silane pre-treatment occasionally creates varying signs of improved longevity and durability of CFRP/steel systems. Silane’s improvement depends heavily on the failure mode, which makes it important to consider silane pre-treatment to various CFRP configurations, to better determine its effectiveness at restricting degradation during moisture exposure. Configurations involving laminates or normal modulus materials may result in more significant improvements from silane pre-treatment as they experience more interfacial and cohesion failures. To further validate the use of silane, it may be required to quantify the proportion of bond strength provided by both the mechanical and chemical components of pre-treated adhered CFRP/steel systems. The portion provided by chemical bonding, for certain CFRP configurations, may pre-determine the potential effectiveness of silane to provide improved adhesion.
- Experiments involving silane pre-treatment also suggested hydrolysis occurred from moisture ingress during environmental submergence. However, this phenomenon requires investigative confirmation by examining the chemical composition of pre-treated steel surfaces, after submergence, to determine if hydrolysis has definitively taken place. This will require the development of a technique to successfully remove residual adhesive from the steel surface, or utilise CFRP systems that undergo steel/adhesive interfacial failure. The surface must then be examined as soon as possible to limit the chemical changes transpiring from atmospheric exposure.
- With environmental exposure occasionally causing levels of debonding it may be applicable to investigate the formation of the debonding region of CFRP/steel systems caused by environmental conditioning and fatigue loading.
- Analytical models require further development to incorporate the significant number of variables related to industrial applications. For example, as failure modes can potentially change as a result of environmental degradation, their fatigue performance significantly alters. Hence, the enhanced model would benefit from a further modification that integrates expected failure modes of CFRP configurations to accurately mimic the reduced performance.

## 6. Summary

This extensive literature review has highlighted weaknesses in understanding of performance of CFRP/steel systems, most importantly in understanding the combined effects of environmental conditioning and mechanical loading. The specific areas of research that require attention and need to be investigated are:

- The galvanic interaction of CFRP/steel systems and their potential to create areas of isolated pitting, which can become high stress regions and the site of premature structural failure.
- The combined effects of environmental exposure and fatigue loading on CFRP/steel joints. Primarily investigating the effect of fatigue stress range, number of applied cycles as well as the exposure temperature and duration. The influence of these variables will allow the design, performance and durability of such bonds to be better understood under industrial conditions.
- The use of adhesive modifiers and chemical primers to restrict the amount of bond degradation witnessed after environmental exposure and fatigue. With durability of such systems being a big threat to their implementation bond strength optimisation is a key to their success.
- The fatigue performance of CFRP repaired steel exposed to environmental conditioning, utilising techniques that proved successful in previously conducted CFRP/steel joint investigations.
- The theoretical prediction of the fatigue life of CFRP repaired steel incorporating the influences of environmental exposure on existing fracture mechanics theories.

## Figures and Tables

**Table 1:** Bond test methods and the highlights of the previous studies.

Steel Type	Carbon Fibre and Carbon Fibre Reinforced Polymer (CFRP) Specification	Test Method	Highlights	Ref.
Low-alloy steel (16-gauge A242 cold-rolled steel)	Unimpregnated Fibre (in tow-sheet form), C1:20 (weight: 200 g/cm^2^, Tensile strength: 3480 MPa, Tensile modulus: 228 GPa). C1:30 (weight: 300 g/cm^2^, Tensile strength: 3480 MPa, Tensile modulus: 228 GPa). C5:30 (weight: 300 g/cm^2^, Tensile strength: 2940 MPa, Tensile modulus: 370 GPa).	Wedge test: The modified wedge-crack specimens (of nominal size 25.4 by 203 mm), which were used to simultaneously evaluate the adhesive steel and adhesive composite bonds.	➢ Owing to the higher density of Cl-30, there are obviously a greater number of interfaces. Consequently, a larger number of potential sites are exposed to environmental attack. ➢ C5-30-based composite system shows a greater degradation compared to C1-30 system under harsher hot water, freeze–thaw, and sea-water environments. The C5-30 system has slightly lower tensile strength (15.5% lower) and a higher (63.6%) tensile modulus.	[1]
A36 steel bar (1/2″ × 1.5″ × 36″)	CFRP plate (0.21″ × 1.44″ × 18″)	Tensile test: A series of increasing tensile loads was applied using an Instron Model 1332 testing machine and the accompanying strain data was recorded. A constant strain rate of 3000 lbs./min was used.	➢ When the force in the steel is increased to yield with respect to specific parameters (e.g., steel substrate and CFRP plate thickness, material properties, etc.), only a certain amount of load will be carried by the CFRP plates. For the specimens tested at 9000 lbs., the maximum force transferred was 1443 lbs. ➢ As the shear stress in the bond attains the maximum shear strength of the adhesive, plastic behaviour results.	[26]
		Fatigue test: A series of small-scale double reinforcement specimens was tested under cyclic loads at a stress range corresponding to the fatigue threshold for common fatigue-sensitive conditions. Double reinforcement specimen is fatigued at a stress range of 12 ksi for 2.55 million cycles.	➢ No sign of debonding of the CFRP plates in any adhesive test group.	
Steel beam (S355J0 (ST 52-3))	CFRP plate (150/2000, width: 50 mm and thickness: 1.2 mm)	Pull-Off Test: Three CFRP plates are gripped inside a friction clamp. Each CFRP plate is pulled using a single-FRP clamp, which is connected to an actuator. The actuators are connected to a hydraulic jack that provides equal pressure for each actuator. An inclined test setup was used due to the deviation of the CFRP plates about 12° in the proposed trapezoidal PUR system.	➢ The triple-FRP clamp slipped at 401.3 kN and the load in the CFRP plates decreased suddenly to 328 kN and remained constant afterward. ➢ For the flat configuration, the rupture occurred in the single-FRP clamps; whereas the slip in the inclined configuration occurred in the triple-FRP clamp.	[29]
		Flexural Test: Three steel beams (one unstrengthened reference and two strengthened with 15% and 31% prestress levels, respectively), were statically tested until failure. A symmetric four-point bending setup is used. The loading span is 1700 mm, whereas the support span is 5000 mm. The test is carried out using a hydraulic testing machine (Pulsator P960) with 250 kN actuator capacity and a force control system.	➢ For unstrengthened beam (B1), the failure mode was yielding in the upper flange followed by a lateral-torsional bulking. ➢ For the beam strengthened by 15% CFRP prestress (B2), the CFRP laminates are initially unstressed; however, they exhibit deflection due to the self-weight. ➢ Increasing the eccentricity (the initial eccentricity e^i^_p_ = 104 mm between the unstressed CFRP plate and the beam) up to 121 mm, resulting a strain of 2320 µm/m in the CFRP plates (15% of the CFRP strength). ➢ The ultimate load-carrying capacity of specimen B2 was increased by more than 23% compared to reference specimen B1. ➢ For the beam strengthened by 31% CFRP prestress (B3), increasing the eccentricity up to 196 mm, resulting in a strain of 4793 µm/m in the CFRP plates (31% of the CFRP strength).	
Steel beam (type IPE 120) yield strain: 1.9 mm/m. Young’s modulus: 199.3 GPa. Yield strength: 383 MPa. Tensile strength: 462 MPa.	CFRP laminates - Normal modulus (150/2000 50/1.4, width: 50 mm, thickness: 1.4 mm, cross-sectional area 70 mm^2^, Young’s modulus: 165 GPa). - High modulus (200/2000 50/1.4, width: 50 mm, thickness: 1.4 mm, cross-sectional area 70 mm^2^, Young’s modulus: 205 GPa). - Ultra-High modulus (Carbolam THM 450 50 × 1.2, width: 50 mm, thickness: 1.4 mm, cross-sectional area 60 mm^2^, Young’s modulus: 440 GPa).	Simply supported four-point bending set-up: Two bearings at the right and left sides of the beam to restrain the vertical and lateral displacements. Only one is free to move longitudinally. Rotations about the longitudinal axis is prevented using fork constraints at both ends of the beam. The test specimens were then loaded vertically using two hydraulic actuators, each having a 100 kN static load capacity. The support span is 1200 mm, while the actuators produce a constant bending moment over a length of 400 mm in the middle of the beam.	➢ Application of the bonded UHM CFRP laminate increased the elastic stiffness of the retrofitted beams substantially (i.e., 14.3% increase compared to the reference unstrengthened beam). ➢ The strains varied along the CFRP laminates and reached their highest value at the constant bending region for the specimen strengthened by the BR system. ➢ The higher the CFRP Young’s modulus, the higher the portion of stresses the CFRP laminate attracted. As a result, using stiffer laminates led to a greater reduction in the tensile stresses at the bottom flange of the steel beams.	[31]
Steel plates (210 mm long, 50 mm wide and 5 mm thick). Mechanical properties (mean elastic modulus: 195 GPa, yield stress: 359 MPa and tensile strength: 484 MPa).	Carbon fibre sheets, MBrace CF 130 (elastic modulus: 240 GPa, ultimate tensile strength: 3800 MPa and ultimate tensile elongation: 1.55%). MBrace CF 530 (elastic modulus: 640 GPa, ultimate tensile strength: 2650 MPa and ultimate tensile elongation: 0.4%).	Fatigue test: number of fatigue cycles (N) ranging from 0.5 million to 6 million at different levels of constant amplitude stress ranges.	➢ When the maximum applied load is <40% of the ultimate static strength there is no fatigue failure observed. ➢ When the maximum applied load is <about 35% of the ultimate static strength, the influence on the bond strength is not significant (less than 10%). ➢ The failure modes were not affected much by the fatigue loading except for those bonded with high modulus CFRP (MBrace CF 530), where fibre fracture extended over more than one cross-section.	[38]
Two steel plates (12 mm thick) are welded to a two rectangular hollow sections (70 mm × 50 mm) of 3 mm thickness.	CFRP plate	Two pull-off tests are carried out on each steel block, one on each of the two thick steel plates.	➢ In some specimens, plate delamination happens after the cohesive debonding crack had propagated over a large part of the interface towards the free end of the FRP plate. In other cases, plate delamination happens first, followed by cohesive failure in the adhesive layer. ➢ At a low load level, the shear stress is the largest at the loaded plate end and then gradually reduces to zero towards the unloaded plate end. As the load increases, the shear stress at the loaded end approaches the local bond strength.	[39]
Steel plates (hot rolled structural steel HA300). The nominal yield stress is 300 MPa. The steel plates are all 20 mm thick and 50 mm width.	CFRP laminates (MBRACEs LAMINATE 460/1500). It is an ultra-high modulus laminate with a nominal elastic modulus of 460 GPa and a nominal tensile strength of 1500 MPa. The laminate thickness is 1.45 mm.	Tension test: Baldwin Universal Testing machine is used (loading rate is 2 mm/min).	➢ Araldite adhesive specimens: the failure mode changed from CFRP delamination to CFRP rupture when the specimen bond length exceeds the effective bond length. ➢ Sikadur adhesive specimens: the failure mode remains mainly cohesive failure independent of the bond length.	[40]

**Table 2:** Fatigue lives and of un-retrofitted and CFRP retrofitted steel beams with initial notches [144].

Stress Range (MPa)	Un-Retrofitted	Retrofitted Beams	Ratio of Fatigue Lives
	Fatigue Life (No. Cycles)	Fatigue Life (No. Cycles)	
207	119,140	379,824	3.2
241	71,278	241,965	3.4
276	35,710	105,345	3.0
310	30,216	75,910	2.5
345	19,068	54,300	2.9

## Data Availability

Data is contained within the article.
